# The Cause and Prevention of Yellow Fever Contained in the Report of the Sanitary Commission of New Orleans

**Published:** 1855-12

**Authors:** 


The Cause and Prevention of Yellow Fever contained in the report of the Sanitary Commission of New Orleans. By E. H. Barton, A.M., M. D., &c., &c.- Philadelphia: Lindsay & Blakiston, 1855.

We have already given our readers the conclusions to which Dr. Barton arrived in reference to the cause and prevention of Yellow Fever, when noticing the report of the Sanitary Commission. In the republication of this portion of the report, Dr. B. has adduced additional facts and arguments to substantiate the positions previously put forth.

We are pleased to see that it is brought out in a more permanent form by the enterprising Philadelphia Publishers.

For sale by D. B. Cook & Co. J.

